# Circular RNA Hsa_circ_0066755 as an Oncogene via sponging miR-651 and as a Promising Diagnostic Biomarker for Nasopharyngeal Carcinoma

**DOI:** 10.7150/ijms.47024

**Published:** 2020-06-15

**Authors:** Jian Wang, Jinyu Kong, Zhong Nie, Diansen Chen, Jun Qiang, Wanqin Gao, Xiaojie Chen

**Affiliations:** 1 Center of Image Diagnoses, The First Affiliated Hospital, and College of Clinical Medicine of Henan University of Science and Technology, Luoyang 471003, China.; 2 Henan Key Laboratory of Cancer Epigenetics; Cancer Institute, The First Affiliated Hospital, and College of Clinical Medicine of Henan University of Science and Technology, Luoyang 471003, China.; 3 Medical College, Henan University of Science and Technology, Luoyang 471003, Henan, China.

**Keywords:** circular RNA, hsa_circ_0066755, nasopharyngeal carcinoma, CNE-1, miR-651, sponge

## Abstract

**Background:** Circular RNAs (circRNAs) represent a class of broad and diversified endogenous RNAs that regulate gene expressions in eukaryotes. *Hsa_circ_006675* has been proven as an important circRNA molecule in nasopharyngeal carcinoma (NPC), however, its function still remains elusive. This study aims to discuss the biofunctions of *hsa_circ_0066755* in NPC.

**Methods:** We detected the expression levels of* hsa_circ_0066755* in NPC patients by quantitative real-time polymerase chain reaction (qRT-PCR), and the corresponding ROC curves were plotted. Functional experiments including CCK-8, colony formation, Transwell assay and Xenograft experiment were conducted. Bioinformatics analysis was performed to seek miRNAs which might have binding sites with *hsa_circ_0066755*. Luciferase reporter assays were finally carried out to verify the binding sites.

**Results:** We found significant increases of *hsa_circ_0066755* in the plasma and tissues of the patients. Moreover, its levels were positively correlated with clinical staging (*P*=0.019). The receiver operating characteristic (ROC) analysis showed that the area under the curves (AUCs) of tissue and plasma* hsa_circ_0066755* for distinguishing NPC from non-cancerous controls were 0.8537 and 0.9044, respectively. Both tissue and plasma *hsa_circ_0066755* testing presented a comparable diagnostic accuracy to the magnetic resonance imaging (MRI). Our in-vitro experiment showed that the overexpression of* hsa_circ_0066755* facilitated the growth, proliferation, clone formation, invasion and migration of CNE-1 NPC cells, while its down-regulation showed completely opposite effects. The xenograft experiment showed that exogenous *hsa_circ_0066755* could significantly enhance the in-vivo tumorigenic ability of CNE-1 cells. Rescue assay further confirmed *hsa_circ_0066755* as a tumor facilitator by sponging *miR-651*.

**Conclusions:** Collectively, this study reported for the first time that* hsa_circ_0066755* played a role of oncogene in NPC and could be used as an effective diagnostic marker for NPC, and that* hsa_circ_0066755* / *miR-651* axis also involved in the progression of NPC.

## Introduction

NPC as a malignant tumor occurs in the nasopharyngeal mucosa, which is commonly reported in southern China and Southeast Asia 1. Most of its pathological types are non-keratinizing, with a high degree of malignancy, aggressive invasion and metastasis profile 2. Owing to the concealed location of the nasopharynx and complicated early symptoms, this disease may be easily missed or misdiagnosed at the early stage 3. A majority of patients have reached the advanced stage by the time they consult a physician; thus, the prognosis is extremely poor 4. Etiological studies have showed that the onset of NPC is associated with EBV infection, genetic susceptibility, dietary habits, and certain environmental and physicochemical factors, of which EBV plays an important role 5, 6. The progression, invasion, and metastasis of NPC are related to abnormal cell movement which is a dynamic process of interactions among various biological behaviors of tumor cells, such as adhesion, degradation, movement and angiogenesis 7, 8. This complicated process is regulated by multiple factors, and involves multiple genes and signaling pathways 9, 10. Therefore, a clear interpretation of the occurrence and development of NPC from the perspective of genetic abnormality has great implications in identifying diagnostic and prognostic indicators, as well as new drug targets.

CircRNA as newly discovered endogenous non-coding RNA encompasses a closed loop structure without a 5'-end cap and 3'-end poly-A tail, primarily consists of exons and is abundant in eukaryotic cells 11, 12. CircRNA is highly conserved and stable, and contains richer transcripts than linear messenger RNA 13. It regulates multiple life activities at the transcriptional and post-transcriptional levels. Studies have shown that circRNA acts as a microRNA (miRNA) sponge to inhibit miRNA activity and block the inhibition of its target by miRNA, thus regulating expressions of other related RNAs 14, 15. In recent years, it has also been proven to be abnormally expressed in a variety of malignant tumors, affect the proliferation, differentiation, apoptosis and invasion of cancer cells, and participate in the onset and development of tumors 13, 16-18. In our previous work, we conducted bioinformatics analysis to analyze the difference of circRNA expression profiles between NPC patients and non-cancer controls that from the GEO database, and found *hsa_circ_0066755* was significantly up-regulated in the NPC tissues. *Hsa_circ_0066755* locates at chr3:108295117-108298535, with a spliced length of 345 base. *Hsa_circ_0066755* was generated from the coding exons of the *KIAA1524* gene (data from circBase, http://circrna.org/). Currently, there is no previous evidence of detailed biofunctions and the corresponding mechanisms of *hsa_circ_0066755* in NPC. The aim of this study was to investigate the expression of *hsa_circ_0066755* in NPC patients and its potential clinical value. The biofunctions and molecular mechanisms in the disease were examined at the cellular and animal levels, aiming to identify new markers and therapeutic targets for future trials in patients.

## Materials and Methods

### Patient specimens

Carcinoma tissues from 30 NPC patients and biopsy tissues from 19 patients with nasal polyps were collected in The First Affiliated Hospital, and College of Clinical Medicine of Henan University of Science and Technology, so were the plasma samples (EDTA-K2 anticoagulant) from 86 NPC newly diagnosed patients and 86 healthy controls. All tissue samples of NPC patients were obtained by biopsy or surgery for the first time and confirmed by histopathological examination. The tissues and plasma samples were immediately frozen at liquid nitrogen or -80 °C. The tumor stage classification was guided by the criteria of the 2017 edition for staging of nasopharyngeal carcinoma in China (The Chinese 2008 expert consensus on staging revision of nasopharyngeal carcinoma). This study was carried out with the approval from the Ethics Committee of The First Affiliated Hospital, and College of Clinical Medicine of Henan University of Science and Technology. Written informed consent was obtained from all participants. This study was conducted in accordance with the Declaration of Helsinki.

### Cell culture and transfection

CNE-1 and CNE-2 cell lines were preserved in our laboratory and cultured in high glucose DMEM medium containing 10% FBS (Hyclone). HEK-293a cell line was purchased from Chinese Academy of Sciences (Shanghai, China) and cultured in RPMI-1640 culture solution containing 10% FBS. *Hsa_circ_0066755* sequence was obtained from the University of California Santa Cruz (UCSC) database (http://genome.ucsc.edu/index.html), connected to the PLCDH-cir vector and packaged as a lentiviral recombinant vector. The Si-circ_0066755 shRNA was also packaged as a lentiviral vector. The target cells were transfected with miR-651 mimics or inhibitor via Lipofectamine™ 2000 (Thermo Fisher SCIENTIFIC) after completing synthesis.

### qRT-PCR

Total RNA extracted from tissue and plasma was reversely transcribed into cDNA (Promega, USA) using RNAprep pure Tissue Kit (TIANGEN, China) and miRNeasy Serum/Plasma Purification Kit. RNA was isolated from the nucleus and cytoplasm using PARIS^TM^ Kit (Life Technologies, USA). The junction of* hsa_circ_0066755* was cloned using the following divergent primers: Forward: '5- GCACTTTTCTTCATGTCTTCACCA-3', reverse: '5- ACTATGGGCCAACAAGGTGAT-3'. The target gene was amplified by FastStart Universal SYBR Green Master (ROX) Kit (Roche, Switzerland). Each sample was analyzed 3 times. *U6* (Forward: '5- CTACCAACACTGTAGAGGAGCC-3', reverse: '5-GCCTCGAAGCTCTCGGTCAT-3') and *GAPDH* (Forward: '5- GGAGCGAGATCCCTCCAAAAT-3', reverse: '5-GGCTGTTGTCATACTTCTCATGG-3') were selected as reference genes for the 2^-ΔΔCt^ relative quantification of *hsa_circ_0066755* in the nucleus and cytoplasm 19. The median expression was used as a cutoff point for grouping the expression level of plasma *hsa_circ_0066755* as “Low” and “High”.

### Cell viability assay

Cells in the logarithmic growth phase were harvested, and 2,000 cells were added into each well of a 96-well culture plate. The total volume was 100 μL, and there were 2 parallel wells for each group. After incubation for 24, 48, 72 and 96 h, 10 μL CCK-8 solution (DOJINDO) was added to each well before another 2h incubation. The optical density (OD) of each well at 450 nm was measured by an ELIASA. The experiment was repeated three times to obtain the average.

### Colony formation assay

Cells in the logarithmic growth phase were harvested and inoculated in a 6-well plate at a density of 800 cells/well. Then these cells were placed in a 37℃, 5%CO_2_ atmosphere, and statically cultured for 2 to 3 weeks until macroscopic clonal cell clusters appeared in the culture dish. Acetic acid/methanol (1:3) of 5 mL was added for fixation for 15 min. The cells were stained with Giemsa staining solution for 20 min and photographed under microscope. The number of cell colonies was counted, and a cluster containing ≥ 50 cells was considered as a clone cluster.

### Wound healing assay

Cells in the logarithmic growth phase were harvested and inoculated into a 6-well plate, and 5×10^5^ cells were added into each well. The cells were placed in a 37 °C, 5% CO_2_ atmosphere overnight and cultured until the fusion rate reached above 90%. A sterile pipette tip (200 μL) was used to perpendicularly scratch cells along a ruler. The migrated cells were washed with PBS and photographed at 0 and 48 h to calculate the number of migrated cells.

### Transwell assay

Invasion experiment was performed by diluting Matrigel (Corning) at the ratio of 1:8 to cover the membrane (upper compartment) of Transwell chamber. Then the chamber was placed at 37 °C for 30 min to allow Matrigel to polymerize into gel (Matrigel may be saved for the migration experiment). The cell density was adjusted to 5×10^5^/mL. Cell suspension (100 µL/well) were added to the upper compartment, and 500 µL 20% FBS containing culture solution was added in the lower compartment of the 24-well plate for chemotaxis before culture in a humidified atmosphere of 5% CO_2_ at 37 °C for 24 to 48 h. The cells and gel in Transwell were scraped off, fixed in 4% paraformaldehyde for 10 min, stained with 0.1% crystal violet for 30 min, and photographed under microscope to count the number of cells in at least 5 different fields of view.

### Immunoblotting

In brief, total protein was extracted by RIPA lysis buffer (Beyotime), and was separated by 12% gel electrophoresis at constant pressure of 80V. The separated proteins were transferred to the PVDF membrane, and were blocked with TBST buffer containing 5% milk powder. The dilutions of the primary antibodies were: rabbit monoclonal Anti-Bcl-2 antibody (Abcam; 1:500), anti-caspase-3 (Beyotime; 1:500), and mouse monoclonal anti-human β-actin (Beyotime; 1:1000). Goat anti-rabbit IgG (1:10000) labeled with horseradish peroxidase (HRP) was utilized as the secondary antibody. ECL luminescent solution was added for the final imaging. Quantity ONE software was used for relative quantification of the proteins.

### Xenograft experiment

The *in-vivo* study was carried strictly following the protocols of Guide for the Care and Use of Laboratory Animals of the NIH (Bethesda, MD). Six-week-old BALB/C nude mice with a weight of 18-20 g were selected. The cell concentration of each group was adjusted to 2×10^7^ cells/mL, and 100 μL cells were subcutaneously inoculated into the middle portion of the inguinal region of each mouse. Subcutaneous tumor was observed around 1 week after inoculation. According to the tumorigenic ability of CNE-1 cells, tumor weights were measured at the end of the fourth week of the experiment. This study was approved by the Ethics of The First Affiliated Hospital, and College of Clinical Medicine of Henan University of Science and Technology.

### Dual luciferase reporter assay

MiRNA molecules and binding sites interacting with *hsa_circ_0066755* were predicted by Circular RNA Interactome (https://circinteractome.nia.nih.gov/). The wild-type (wt) and mutant (mut) *hsa_circ_006675*5 sequences were separately designed and cloned into the pmirGLO vector (Promega). Its recombinant vector and miR-651 mimics were co-transfected to HEK-293a cells via Lipofectamine™ 2000 (Thermo Fisher SCIENTIFIC) according to its protocol. The cells were lysed after 24 h of culture and luciferase activity was measured.

### Statistical analyses

Statistical analysis was performed using SPSS 16.0 software. Measurement data were expressed as mean±standard deviation (SD). If the assumptions of normality and homoscedasticity were satisfied, Student's *t*-test was conducted to compare the differences between the two groups. Correlations between *hsa_circ_0066755* expression and clinical pathological characterstics were analyzed by *chi*-square test or Spearman correlation analysis. The diagnostic value was evaluated by drawing a ROC curve. A *P* value of < 0.05 was considered for statistical significance.

## Results

### Hsa_circ_0066755 was up-regulated in NPC patients

**Figure 1:**
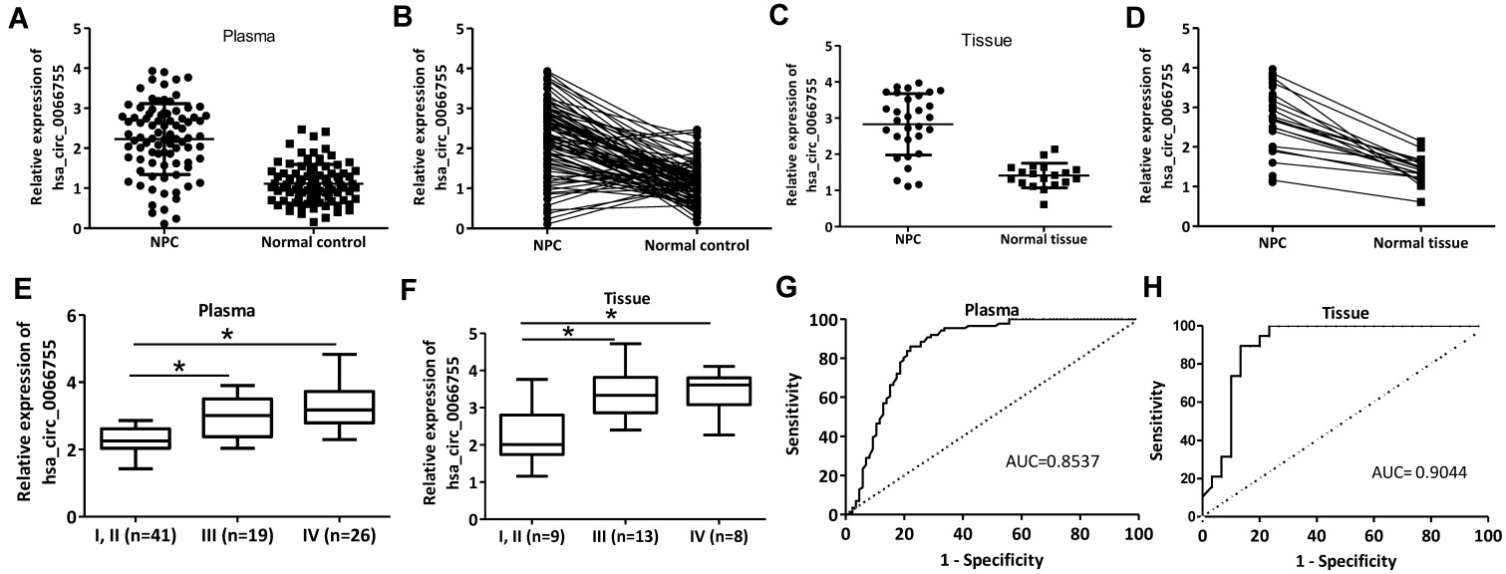
Plasma and tissue levels of *hsa_circ_0066755* in NPC patients and the clinical significance. (A,B) Plasma and (C, D) tissue levels of *hsa_circ_0066755* in NPC patients detected by qRT-PCR. (E, F) Plasmic or tissue levels of *hsa_circ_0066755* were higher in stage III and IV NPC patients than those in stage I and II patients. The plotted ROC curves of (G) plasma and (H) tissue *hsa_circ_0066755* in the diagnosis of NPC. *P < 0.05.

Plasma levels of* hsa_circ_0066755* in 86 newly diagnosed NPC patients and 86 healthy controls were tested by qRT-PCR. As a result, the NPC group presented a significantly higher level of hsa_circ_0066755 than the control group (Figure 1A and 1B).* Hsa_circ_0066755* levels in 16 pairs of NPC tissues were determined, which also revealed a significant up-regulation in the tissues (Figure 1C and 1D). Furthermore, stage I and II NPC patients showed lower plasma and tissue levels of *hsa_circ_0066755* than those in stage III and IV (Figure 1E and 1F). Correspondingly, correlation analysis confirmed that plasma* hsa_circ_0066755* expression was positively correlated with clinical staging (*P*=0.019) (Table 1). ROC analysis showed that the AUCs of plasma and tissue *hsa_circ_0066755* for distinguishing non-cancerous controls from NPC subjects were 0.8537 and 0.9044, respectively (Figure 1G and 1H). We followed up 24 of 86 patients (including all 16 tissue donors) for more than 3 months. The results showed that 23 patients with positive MRI findings were diagnosed as NPC by histopathological examination. The diagnostic accuracy of MRI and plasma and tissue *hsa_circ_0066755* was 95.83% (23/24), 83.33% (20/24) and 87.50% (14/16) (Table 2), suggesting that plasma and tissue *hsa_circ_0066755* were very useful in the diagnosis of NPC.

### Overexpression of *hsa_circ_0066755* promoted the proliferation and invasion of CNE-1 cells

**Figure 2:**
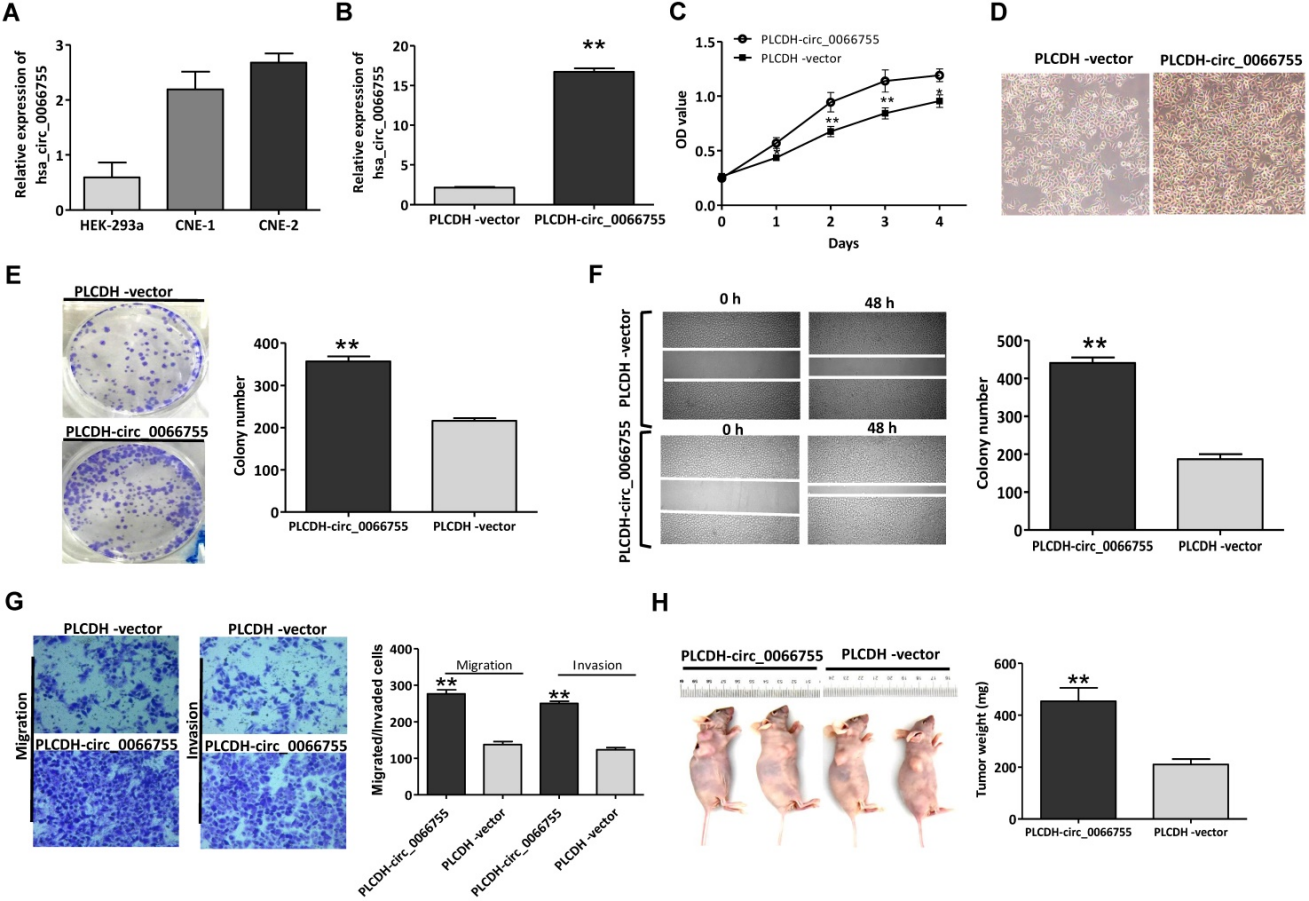
Up-regulation of* hsa_circ_0066755* promoted the growth, proliferation, invasion and migration of CNE-1 cells. (A) Basal levels of *hsa_circ_0066755* in HEK-293a, CNE-1 and CNE-2 cell lines. (B) The expression of total *hsa_circ_0066755* in CNE-1 cells following infection. (C, D) Cell viability assessed by CCK-8 assay, and cells morphology and growth condition was imaged at 72h following infection (×200). (E) Colony formation assay. (F) Wound healing migration assay. Migrated cells were counted at 48 h after transfection. (G) Transwell invasion assay. Migrated or invaded cells were counted at 48 h following transfection with or without Matrigel. (H) Xenograft experiment. Tumors were weighed at the endpoint of the experiment. *P < 0.05, **P < 0.01* vs.* PLCDH-vector group.

Results of qRT-PCR showed that CNE-1 and CNE-2 cells presenting certain levels of *hsa_circ_0066755*, which were significantly higher than those in HEK-293a cells (Figure 2A). CNE-1 cells that moderately expressed* hsa_circ_0066755* were selected as the target cells. After infected with PLCDH-circ_0066755, *hsa_circ_0066755* levels in CNE-1 cells increased by 7.78 folds (Figure 2B). The CCK-8 assay showed that up-regulated exogenous *hsa_circ_0066755* expression could significantly promote the growth of CNE-1 cells (Figure 2C and 2D). The clone formation experiment showed that the number of cloned cells in the PLCDH-circ_0066755 group was significantly higher than that in the PLCDH-vector group (*P* < 0.01) (Figure 2E). Scratch wound healing and Transwell chamber experiments showed that the number of invasive and migrated cells in the PLCDH-circ_0066755 group was significantly higher than that in the PLCDH-vector group (Figure 2F and 2G). These results suggested that the overexpression of *hsa_circ_0066755* promoted the growth, clone formation, invasion and migration of CNE-2 cells. Thus, we further designed an *in vivo* experiment. The tumor formation experiment of nude mice showed that the PLCDH-circ_0066755 group presented significantly heavier weights of tumors and evidently stronger tumorigenic abilities than the PLCDH-vector group (Figure 2H).

### Hsa_circ_0066755 silencing inhibited the proliferation and invasion of CNE-1 cells

**Figure 3:**
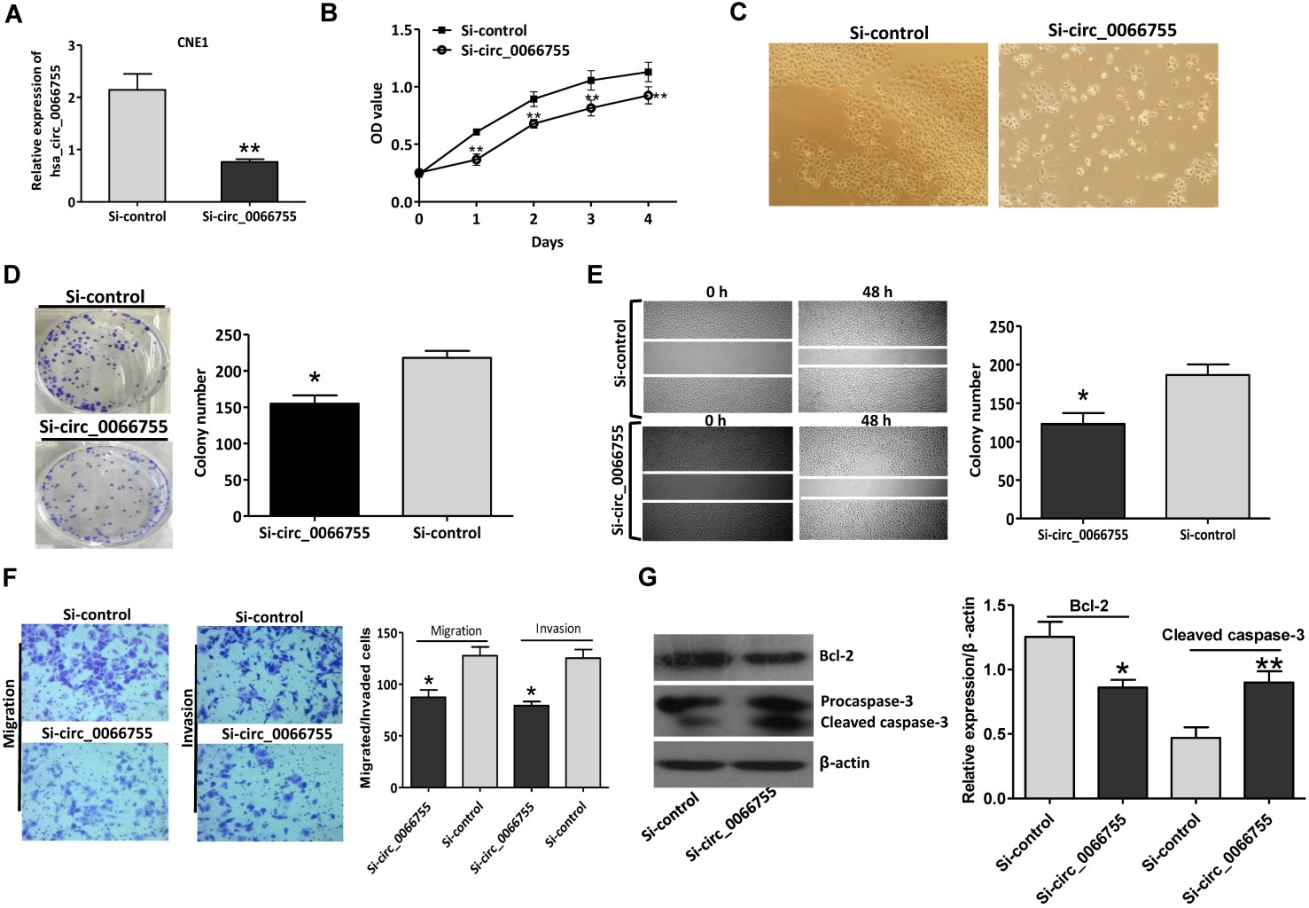
*Hsa_circ_0066755* silencing inhibited the proliferation and invasion of CNE-1 cells. (A) The expression of *hsa_circ_0066755* in CNE-1 cells following transfection with the si-hsa_circ_0066755 vector. (B) Cell viability assessed by CCK-8 assay. (C) Morphological changes of CNE-1 cells after infected with the Si- circ_0066755 shRNA vector at 72h. (D) Colony formation assay. (E) Wound healing migration assay. Migrated cells were counted at 48 h after silencing. (F) Transwell invasion assay. Migrated or invaded cells were counted at 48 h after transfection with or without Matrigel. (G) Expression of apoptosis related proteins detected by immunoblotting. *P < 0.05, **P < 0.01* vs.* Si-control group.

Results of qRT-PCR indicated that after silencing levels of* hsa_circ_0066755* in CNE-1 cells were significantly down-regulated (Figure 3A). The CCK-8 test showed that down-regulating endogenous *hsa_circ_0066755* expression could obviously inhibit the growth of CNE-1 cells (Figure 3B and 3C). As illustrated in Figure 3D, the number of cloned cells in the Si-circ_0066755 group was evidently lower than that in the Si-vector group (*P* < 0.05). Scratch-wound healing and Transwell chamber experiments revealed that the number of invasive and migrated cells in the Si-circ_0066755 group was much lower than that in the Si-vector group (Figure 3E and 3F). Moreover, immunoblotting showed that expression of Bcl-2 protein in Si-circ_0066755 group were down-regulated, along with an activation of procaspase-3, suggesting that *hsa_circ_0066755* silencing may induce apoptosis in CEN1 cells (Figure 3G). Collectively, these results suggested that down-regulating endogenous *hsa_circ_0066755* expression could inhibit the growth, clone formation, migration and invasion of CNE-1 cells.

### Hsa_circ_0066755 regulated NPC progression by sponging miR-651

**Figure 4:**
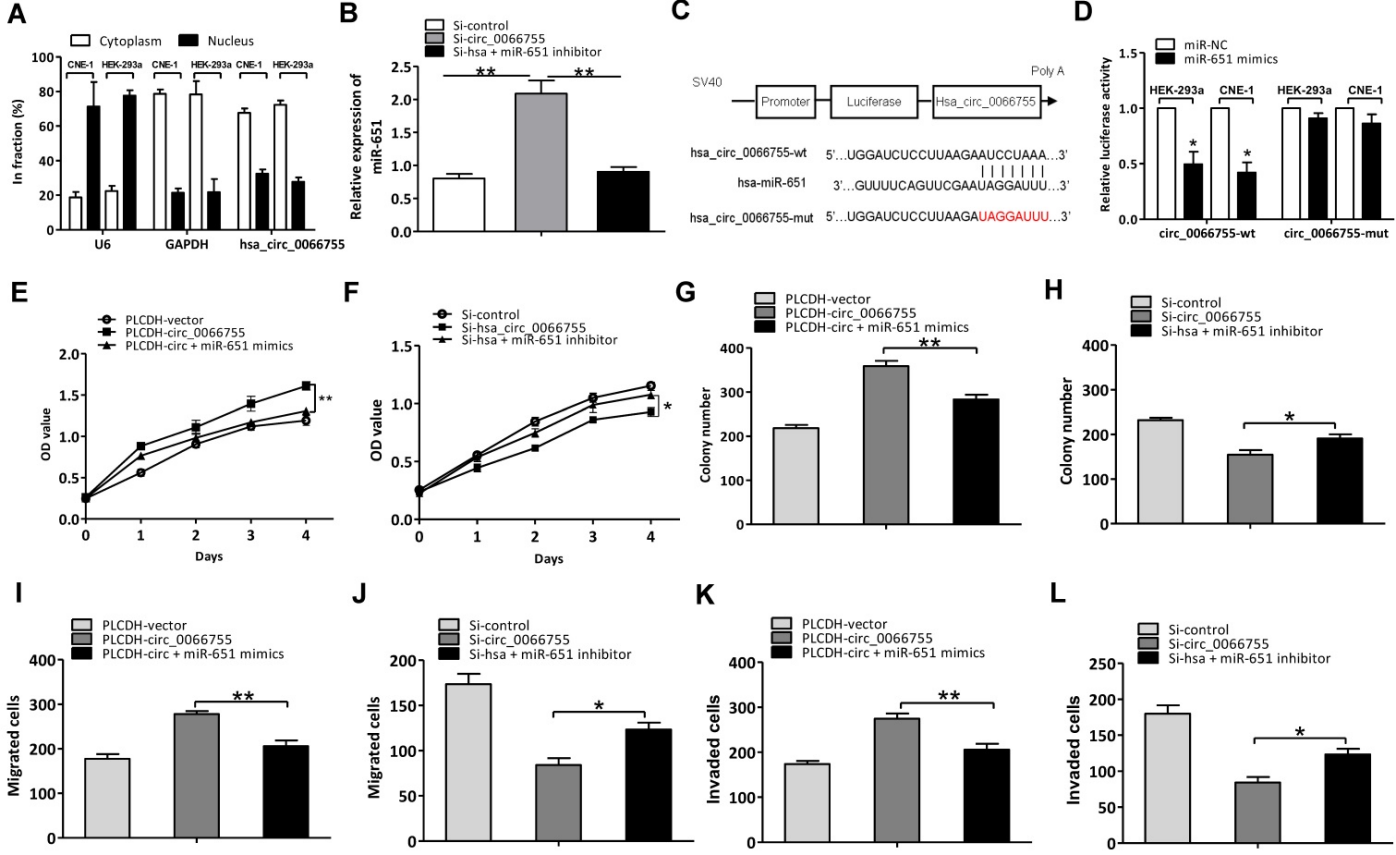
*Hsa_circ_0066755* acted as the *miR-651* sponge to regulate NPC progression. (A) Distribution of *hsa_circ_0066755* in nuclear and cytoplasmic fractions of CNE-1 cells. (B)* Hsa_circ_0066755* silencing could increase *miR-651* expression. (C, D) Luciferase assay of HEK-293a and CNE-1 cells co-transfected with the *miR-651* mimic and luciferase reporter containing hsa_circ_0066755-wild-type sequences (cirRNA-wt) or -mutant sequences (cirRNA-mut). (E, F) Cell viability of CNE-1 cells assessed by CCK-8 assay. (G, H) Colony formation assay. (I, J) Wound healing migration assay. (K, L) Transwell invasion assay. *P < 0.05, **P < 0.01.

As *hsa_circ_0066755* has been proven to be located in the cytoplasm of CNE-1 cells, we assumed that *hsa_circ_0066755* possibly acts as the sponge of miRNA (Figure 4A). Based on the predicted results in our bioinformatic analysis, 17 kinds of miRNAs potentially interacted with hsa_circ_0066755 (Table 3). Four were identified to have significant inhibitory effects on luciferase activity using the luciferase reporter gene screening system for miRNA target genes, of which *miR-651* showed superior inhibitory effects over others. To verify the effects of* hsa_circ_0066755* on* miR-651*, we silenced the gene by transfecting si-hsa_circ_0066755 shRNA vectors into CNE-1 cells. As a result, *miR-651* expressions obviously increased (Figure 4B). Besides, the dual luciferase reporter assay revealed that luciferase activity in CNE-1 and HEK-293a cells co-transfected with *miR-651* mimics in the hsa_circ_006675-wt group was significantly lowered compared with the negative control group, while the hsa_circ_006675-mut group presented nonsignificant changes in luciferase activity (Figure 4C and 4D). Using the* miR-651* mimic and inhibitor, we successfully proved the positive and negative effects of the interaction between* hsa_circ_0066755* and m*iR-651* on the biological characteristics of CNE-1 cells. The CCK-8 test alongside the clone formation test, the scratch assay and the Transwell experiment showed that the circ_0066755+mimic group presented weaker growth of CNE-1 cells, a lower number of cloned cells and weaker cell invasion and migration than the circ_0066755 group; however, the Si-circ_0066755+inhibitor group revealed evidently stronger growth of CNE-1 cells, a higher number of cloned cells and stronger cell invasion and migration than the Si-circ_0066755 group (Figure 4E to 4L).

## Discussion

Though the onset is regional, NPC owns a high incidence in an Asian population 1, 4. Because of the non-specific symptoms at the early stage, most initial confirmed cases have entered the late stage with a high likelihood of distal metastasis and a high risk local recurrence 2, 3, 5. Better understanding of mechanisms behind its onset and development and identification of reliable markers for NPC are essential to the clinical diagnosis and treatments. CircRNA as a special class of non-coding RNA molecules has been a latest hotspot 11, 12. It may act as a constituent of competitive RNA (ceRNA) to inhibit the activity of miRNA, regulating gene transcription, translation and other functions thereby 20-22. This study has explored the biofunctions of *hsa_circ_0066755* in NPC and the corresponding mechanisms, and discussed its potential clinical value as a diagnostic biomarker for NPC.

At present, very few studies have focused on circRNAs in NPC. Some have reported that circMAN1A2 expression is elevated in NPC tissue and is highly effective in its diagnosis (AUC = 0.91) 23. Shuai *et al*. have found that highly expressed *circRNA_0000285* in NPC is associated with tumor size, tumor cell differentiation, lymph node metastasis, distant metastasis, etc., which may be used as an independent prognostic indicator 24. This study shows that plasma and tissue *hsa_circ_0066755* expressions evidently increase in NPC patients and are positively correlated with tumor progression and TNM staging, which in turn are highly effective in the diagnosis of NPC with AUCs of 0.8537 and 0.9044, respectively. Moreover, plasma* hsa_circ_0066755* is highly consistent with MRI in terms of the diagnosis of NPC. An easier access to blood samples with a less-invasive sampling process has made plasma *hsa_circ_0066755* as one of the promising diagnostic indicators of NPC.

Therefore, to the best of our knowledge, we have for the first time explored the biofunctions of *hsa_circ_0066755* in NPC, pro and con. It is found that exogenous *hsa_circ_0066755* can significantly improve the growth, clone formation, and invasion and migration of CNE-1 cells, while its down-regulation leads to the opposite results. Our *in-vivo* experiment further indicates that *hsa_circ_0066755* can improve the tumorigenic ability of CNE-1 cells in nude mice. To sum up, this study proves that *hsa_circ_0066755* functions as an oncogene in NPC, which correlates with its clinical progression. A study by Zhu *et al*. suggests that down-regulating *circ-ZNF609* expression in NPC 5-8F and HONE-1 cells can significantly repress cell proliferation, invasion and migration 25, which is similar to our findings.

Studies have shown that circRNA can regulate expressions of its target genes via sponging miRNA, which has been proven in multiple malignant tumors and their progressions 26-29. The qRT-PCR analysis in our study has confirmed that *hsa_circ_0066755* is mainly distributed in the cytoplasm of CNE-1 and HEK-293a cells. Thus, we have assumed that *hsa_circ_0066755* may play the role as a miRNA sponge. Based on the bioinformatic analysis, we have predicted that there are 46 potential miRNA molecules interacting with* hsa_circ_0066755*. Through the screening of the luciferase reporter gene screening system for miRNA target genes, 4 present significantly inhibitory effects on luciferase activity, and *miR-651* is the most prominent factor. To decrease the confounding factors, we selected human HEK293a and CNE-1 cells for the study of *hsa_circ_0066755* and *miR-651* interaction. Our study further verified that *hsa_circ_0066755* interacts with *miR-651* and they can affect the biological characteristics of CNE-1 cells.

Nevertheless, this study still has limitations, including a small sample size, only one cell line used for verifying cell functions, a lack of deep investigation of the possible apoptosis effects, and failure to confirm the* hsa_circ_0066755* silencing experiments in-vivo, etc. Our research team will constantly improve the experiments above to overcome these limitations in the future.

In conclusion,* hsa_circ_0066755* expression is up-regulated in NPC and is associated with the progression of NPC. Plasma and tissue* hsa_circ_0066755* is very effective in the diagnosis of NPC, of which plasma* hsa_circ_0066755* can be promoted as a diagnostic biomarker due to an easier access to blood samples than tumor tissues. Besides, *hsa_circ_0066755* involves as the *miR-651* sponge in the growth, proliferation, invasion and migration of NPC cells.

## Figures and Tables

**Table 1:** Clinical characteristics of plasmic* hsa_circ_0066755* expression in NPC

Clinicopathological features	Total (n=86)	Low (n=33)	High (n=53)	χ^2^ value	*P* value
**Gender**				0.029	0.864
Male	66	25	41		
Female	20	8	12		
**Age (years)**				0.969	0.325
≤45	37	12	25		
>45	49	21	28		
**Clinical stage**				5.469	0.019
I-II	41	21	20		
III-IV	45	12	33		
**Pathological type**				1.625	0.202
Nonkeratinizing	85	32	53		
Keratinizing	1	1	0		
**Lymphatic metastasis**				1.903	0.168
Yes	24	12	12		
No	62	21	41		

**Table 2:** Diagnostic accuracy of plasma and tissue hsa_circ_0066755 and MRI in confirming NPC

Groups	Total size	Missed diagnosis (%)	Misdiagnosis (%)	Diagnostic accuracy (%)
Plasmic* hsa_circ_0066755*	24	4 (16.67)	0	20 (83.33)
Tissue *hsa_circ_0066755*	16	2 (12.5)	0	14 (87.5)
MRI	24	0	1 (4.17)	23 (95.83)

**Table 3:** The predicted miRNAs that may target with *hsa_circ_0066755*

Predicted miRNAs	Binding site with *hsa_circ_0066755*	Context+ score percentile
*hsa-miR-1224-3p*	5'…UCCACCC…3'	96
*hsa-miR-1243*	5'…UAGGUCA…3'	98
*hsa-miR-1261*	5'…AAUAGGU…3'	97
*hsa-miR-140-3p*	5'…GACACCA…3'	98
*hsa-miR-323-3p*	5'…CAUUACA…3'	NA
*hsa-miR-361-3p*	5'…ACCCCC…3'	92
*hsa-miR-488*	5'…GAAAGU…3'	87
*hsa-miR-545*	5'…AACGAC…3'	93
*hsa-miR-579*	5'…UUUACU…3'	90
*hsa-miR-580*	5'…UAAGAGU…3'	99
*hsa-miR-587*	5'…UACCUU…3'	87
*hsa-miR-606*	5'…UCAUCAA…3'	97
*hsa-miR-624*	5'…UGGAACA…3'	94
*hsa-miR-651*	5'…UAGGAUU…3'	99
*hsa-miR-885-5p*	5'…AUUACC…3'	89
*hsa-miR-890*	5'…GGUUCA…3'	92
*hsa-miR-936*	5'…GAUGAC…3'	89

## Abbreviations

AUC: area under the curve
ceRNA: competitive RNA
circRNAs: circular RNAs
miRNA: microRNA
MRI: magnetic resonance imaging
NPC: nasopharyngeal carcinoma
qRT-PCR: quantitative real-time polymerase chain reaction
ROC: receiver operating characteristic
